# Supplementary material for: Are carbon emissions trading and green financial instruments synergistic? -Comprehensive quantitative research based on content analysis
Source: PLoS One. 2024 Mar 7;19(3):e0298601. doi: 10.1371/journal.pone.0298601 (PMC10919601; doi:10.1371/journal.pone.0298601)
Supplement: S1 Data — (ZIP) [file pone.0298601.s001.zip › Data/地级市Z_F工作报告.zip!/╔┬╬≈/═¡┤¿╩╨/└┤╫╘╣1⁄2╓┌║┼í╛┬φ┐╦╩2╛▌═°í┐_2015.docx]

各位代表：

　　现在，我代表市人民政府，向大会报告工作，请予审议，并请市政协各位委员提出意见。

　　一、市十五届人大五次会议确定任务完成情况

　　去年，面对复杂严峻的宏观环境，在省委、省政府和市委的坚强领导下，我们深入贯彻落实党的十八大和十八届三中、四中全会精神，牢牢把握稳中求进的工作总基调，用好一切有利于铜川发展的机遇，倾注力量补短板，精准发力破难题，保持定力促转型，突出“三重”抓“三区”，较好地完成了市十五届人大五次会议确定的主要目标任务。

　　（一）着力转变发展方式，经济持续稳健增长。在传统支柱产业受到严重冲击的情况下，调结构、转方式、稳增长，经济运行处于合理区间。实现生产总值340.42亿元，增长11%，高于全省1.3个百分点；地方财政收入22.06亿元，同口径增长14%，高于全省0.4个百分点；城乡居民收入分别为27237元、9169元，增长11.2%、12.6%，高于全省2.2和0.8个百分点。居民消费价格累计上涨1.8%。

　　（二）优化环境服务保障，项目建设明显加快。实行驻工地专员等制度，着力解决项目推进“卡脖子”问题。市级重点项目完成投资162.2亿元，新开工项目30个，23个建成投产投运。40个转型骨干项目、28个节能减排示范项目有序推进。铭帝铝材、现代陶瓷、美佳华商业广场省考项目，完成投资任务的156.3%。招商引资签约项目208个，到位资金141.81亿元。全社会固定资产投资完成327.63亿元，增长26%。

　　（三）贯彻扶持调控政策，工业经济负重前行。制定了稳工业增长20条措施，在统的36种工业品25种保持增长。建成工业项目13个，新增规模以上企业19户。规模以上工业增加值186.36亿元，增长12.8%。工业品产销率95.8%。装备制造、食品加工、医药保健等接续产业增加值增长15.8%，高于传统支柱产业4.1个百分点。理顺工业园区财税、融资体制，激发园区创业活力，园区产值占到全市工业的61%。

　　（四）文化旅游项目引领，服务消费增势强劲。开展了唱响铜川、书香铜川群众文化活动，“两馆一站”免费开放，政府购买公共演出服务200场，文化产业增加值增长12.3%。66个旅游项目完成投资8.8亿元。照金香山被评为国家4A级景区，照金国际滑雪场建成运营。药王山景区规划通过评审，亮化工程展现独特魅力。宜君战国（魏）长城遗址入选世界文化遗产中国长城名录。印台区通过省文化先进区验收。市旅游服务中心建成投运。成功举办了首届海峡两岸孙思邈中医药合作与发展研讨会、全国大学生红色旅游线路设计大赛决赛。全年接待游客992.9万人次，旅游总收入53.6亿元，分别增长11.7%、22%。社会消费品零售总额82.55亿元，增长14.1%。新增省级名牌产品3个。服务业增加值96.11亿元，增长9.6%。外贸进出口总额2231万美元，增长34.6%。

　　（五）巩固农业基础地位，现代农业稳步发展。耕地保有量147.3万亩，基本农田保护面积113.7万亩。粮食总产23.39万吨，实现“十一连丰”。农业园区完成投资10.5亿元。农村土地流转2.2万亩，增长33%。引进了昊诚、雅荷等集团，实施农业产业化项目及优美小镇建设。照金现代农业园被列为全国休闲农业与乡村旅游示范点。伊利奶牛养殖一期工程完工。建成海升千亩矮砧现代苹果园，耀州区移寨果园被确定为中（国）哈（萨克斯坦）苹果友谊园。水果总产69.06万吨，增长2.5%。宜君县被授予“中国核桃之乡”。

　　（六）抓精细化网格管理，城市品质不断提升。在城市基础设施及绿化亮化等方面“添大件、置家当”，在徒步街巷现场管理、网格化监督上持续作为。国家卫生城市创建工作通过全国爱卫办综合评估，宜君县被命名为国家卫生县城。新区阳光广场续建工程建成使用。修复城市破损路面、人行道3.2万平方米，新建公厕18座，新增公共绿地60万平方米，完成163栋建筑物亮化。注重在城市建设中融入地域文化符号，不断增量城市元素。新区城市供水管网二期改建工程投入使用。生活垃圾无害化处理率88.6%，污水处理率89.4%。照金、董家河、黄堡、陈炉、彭镇被确定为全国重点镇，黄堡省级重点示范镇和照金、陈炉文化旅游名镇建设加快。1.65万农村居民进城落户，城镇化率62.22%。

　　（七）硬法硬手治污降霾，生态建设成效明显。建立了企业环境信用评价“红黑榜”制度，实施了20家重点企业环保设施提标改造工程。华能照金电厂2号机组超低排放改造达到预期效果。210国道环境综合整治扎实推进。关闭拆除石碴厂、白灰窑59个，拆除燃煤锅炉183台，新建天然气锅炉146台，新增天然气用户1.02万户，城市气化率76%。植树造林13.25万亩，治理水土流失207.9平方公里，森林覆盖率46.5%。万元生产总值能耗下降6.62%，二氧化硫、氮氧化物、化学需氧量、氨氮排放分别削减0.81%、9.68%、1.01%和2%。空气质量优良天数229天。水源地水质达标率保持在98%以上，出市断面水质达到Ⅳ类标准。沮河流域野化放飞的朱鹮生存状态良好，繁育出了铜川籍小朱鹮。在2014年省信息中心幸福城市、宜居城市排名中，我市列全省第二、第三位。

　　（八）兜底补短保障民生，群众生活有所改善。民生支出占公共预算支出的80.3%，新增财力的87.9%用于民生。城镇新增就业1.09万人，登记失业率3.35%。提高了城乡居民基础养老金和丧葬费补贴标准，企业退休人员养老金月均增加187.3元。新农合筹资标准提高到430元，参合率99.3%。为8.3万城乡低保对象发放保障金2.15亿元，为4838名残疾人免费适配辅助器具8300件。开工建设保障房11381套，竣工13450套，1.05万户入住新居。地质灾害隐患点群众搬迁三年计划全面完成，累计搬迁安置群众8626人。解决了2.06万农村居民饮水安全问题，2.16万农村贫困人口实现脱贫。慈善科技扶贫、捐资助学、救灾济困成效显著。耀州区、新区“双高双普”创建和义务教育均衡发展及全市语言文字工作通过省级评估验收，农村义务教育营养改善计划受益学生2.34万人。加大专项整治和依法监管力度，食品药品安全形势稳定向好。“单独两孩”政策平稳实施，实现了国家计划生育优质服务先进区县全覆盖。建成黄堡、照金文体中心和13个广播电视户户通乡镇服务站、61个村体育健身工程。安全生产四项控制指标全面下降，被评为全省安全生产先进单位。严打整治和大巡防工作力度加大，社会治安状况良好，被评为全省社会治安综合治理优秀市。信访秩序不断好转，实现重大活动、重要会议及重点时段“零集访”。

　　（九）破解体制机制障碍，改革创新成效显现。8个领域42项重点改革任务进展顺利，34项全面完成。取消下放行政审批152项，精简率36.5%。2013年度全省县域经济考评中，我市各区县获得“三进一平”的好成绩，王益区获得争先进位奖。在60家规模企业、40个重点项目实行企业收费登记簿制度。完成了交通运输业、部分现代服务业、邮政电信业“营改增”任务。长安银行落户铜川。全市新登记各类市场主体5887户，增长25.9%。非公经济占比48.6%，提高了0.5个百分点。建设市、县、乡、村便民服务大厅，设立便民窗口408个，办结群众申请事项59725件。

　　（十）开展教育实践活动，政府作风效能提升。扎实开展群众路线教育实践活动，及时解决了一批群众反映的突出问题。市级部门财政预决算全面公开，压缩“三公”经费23.4%，精简会议文件23%。清理违规车辆107台、办公用房8393平方米，停建楼堂馆所3个。查处违反八项规定案件52起，处理62人。实施规范性文件三统一制度，切实加强行政执法队伍建设，严肃查处不当执法行为。坚持开门问政，奏响公民代表进政府、大学生假期到政府见习、网民留言办理微博微信发布“政务公开三部曲”。1604名公民代表走进市、县、乡政府，84名公众代表列席政府会议，187名全国各地大学生到政府见习。办理人民网市长留言42件、“市长信箱”1955件，发布政务微博5378条，市政府网站访问量达到2400万人次。办理人大代表建议75件、政协提案240件。

　　双拥共建活动深入开展，妇女儿童事业加快发展，统计、档案、人防、气象、民族宗教、防震减灾等工作都取得了新成绩。

　　各位代表，过去一年的成绩来之不易。这是市委统揽全局、科学决策的结果，是市人大、市政协及社会各界支持监督的结果，更是全市人民团结拼搏、埋头苦干的结果。我代表市人民政府，向全市人民，向关心支持铜川发展的各界人士，表示衷心的感谢！

　　我们也清醒地看到，发展中存在一些困难和问题，政府工作还有不少缺点和不足。主要是：经济下行压力持续加大，传统支柱产业受到重创，出现了前所未有的严峻形势，接续产业占比低，消费是经济发展的短板，产业转型升级任务艰巨；大项目特别是骨干财源项目少，创新驱动和市场主体活力不足；城乡基础设施欠账多，环境治理及生态建设任务重；政府职能转变不到位，办事效率、服务水平有待提高，简政放权力度需进一步加大，法治政府建设和反腐败斗争仍是长期任务。我们一定要深刻认识不足，敢于直面问题，一个一个从严从细梳理，一件一件坚决严肃整改，以实实在在的变化和成效交出一份让人民群众认可的答卷。

　　二、今年工作的总体要求和主要预期目标

　　当前，宏观经济环境正在发生深刻变化，中央作出了我国发展进入新常态的战略判断。铜川转型发展进入攻坚期，既要稳增长，又要促转型；既要抓发展，又要优环境，转型发展越来越难。但机遇更加难得。2月14日，习近平总书记来我市视察，让铜川人长精神，这是对我们的巨大鼓舞和鞭策，更是推进铜川转型发展的强大动力。陕西是西北地区的重要省份，是实施“一带一路”战略的重要节点，正处在追赶超越阶段，国家实施优先推进西部大开发战略，陕西将获得更多支持；中央赋予陕西西部科学发展新引擎、内陆改革开放新高地和中国特色新型城镇化范例等重大使命，给陕西发展带来新机遇；铜川是资源转型和节能减排两个国家级试点城市，延西高速铜川北至黄陵段和铜（川）旬（邑）高速年底通车，西（安）铜（川）城际铁路开工，我市的区位优势更加凸显。我们要用智慧和定力推进产业、城市、社会、体制机制系统转型，让人民群众体面劳动、尊严生活，让更多的人对铜川充满向往。

　　今年政府工作的总体要求是：认真学习贯彻习近平总书记来陕视察重要讲话精神，全面贯彻落实党的十八大和十八届三中、四中全会、中央经济工作会议精神，紧扣“四个全面”的战略布局和“五个扎实”重要要求，贯彻落实省委、省政府及市委的决策部署，坚持稳中求进的工作总基调，继续以转型发展为统揽，以提高发展质量和效益为中心，系统推进稳增长、促改革、调结构、惠民生、防风险各项工作，强化创新驱动，着力破解转型中遇到的困难和问题，促进经济平稳健康发展、社会和谐稳定，力争全面完成“十二五”规划目标任务，加快建设经济强、文化兴、生态美的全国知名休闲养生城市。主要预期目标是：生产总值增长10%左右，地方财政收入同口径增长12%，全社会固定资产投资增长22%，社会消费品零售总额增长13%，服务业增加值增长9%，城镇登记失业率控制在4%以内，城乡居民收入分别增长10.6%、10%，居民消费价格涨幅3%左右，万元生产总值能耗下降3.3%，二氧化硫、氮氧化物、化学需氧量、氨氮排放分别削减0.3%、6%、1%和2%。

　　加快转型发展是解决铜川一切问题的基础和关键，也是一场持久战。缩小差距、促进就业、改善民生、引领社会信心，都必须保持一个合理的增长速度。今年这样的安排，是基于转型不失速的考虑，旨在实现存量调结构腾空间、增量优结构扩空间，加快培育新的增长点；更是基于实现“十二五”规划目标，为“十三五”打下坚实的基础。我们一定要保持定力，主动作为，切实做到调速不减势、量增质更优，在新常态下开创铜川工作新局面！

　　三、今年的主要工作任务

　　（一）坚持抓项目保重点，促进经济平稳增长

　　加快重点项目建设。坚持以项目带动投资，以投资促进发展，加快100个市级重点项目建设，完成投资150亿元。继续抓好“铜七条”落实，确保美鑫循环经济产业项目组团、陕汽零部件加工基地三期、阿庄风电、210国道川口至耀州段改扩建等20个项目建成投产投运，西北耐物流园、川口至陈炉旅游公路等41个项目开工建设，16个前期项目取得实质进展。

　　保持工业稳定运行。认真落实工业稳增长各项政策措施，注重发挥企业家才能，盘存量、抓增量，降成本、提效益，防止传统支柱产业严重下滑。加强经济运行调度和分析监测，定向施策，对重点行业、骨干企业“一业一策、一企一策”服务。抓住国家恢复煤炭进口关税的有利时机，努力开拓煤炭市场，支持华能照金电厂开展直供电试点，促进煤炭就地转化。改进水泥营销策略，开拓西安、陕北及省外销售市场，提高铜川水泥市场占有率。坚持环保优先，产城融合，加快工业园区基础设施建设，完善服务功能，促进项目落地，推动产业聚集发展。做好工业新建项目达产达效和现有企业改造升级。全年新增规模以上工业企业10户，规模以上工业增加值力争增长14%。

　　努力扩大消费需求。积极推进收入分配制度改革，千方百计提高城乡居民收入。督促企业严格执行工资集体协商制度，适时提高企业工资指导线。调整机关事业单位基本工资标准，增加离退休人员离退休费，建立县以下机关公务员职务与职级并行制度。完善兼顾各类人员的社会保障救助标准正常调整机制，保障干部职工正常福利待遇，落实带薪休假制度。加快美佳华商业广场等综合体建设，大力发展特色商业街区、餐饮街区和品牌店。健全农村流通和社区商业服务网络。培育汽车、信息、休闲旅游等消费热点，促进养老家政健康消费，规范快递物流服务，加快发展电子商务等新型业态。积极落实提高住房公积金使用率的政策措施，引导房地产健康发展。健全社会信用体系，强化质量安全监管，严厉打击制假售假、商业欺诈、非法融资等违法行为。

　　加大招商引资力度。瞄准中省投资方向，突出传统产业升级、基础设施、文化旅游、养生保健等重点领域，谋划、包装、储备一批事关我市转型发展的大项目、好项目，把发展机遇和政策优势转化为实实在在的项目。广泛开展以商招商、专业招商、定向招商、产业链招商，提升招商引资质量。在公用事业、环境保护等领域，推出一批鼓励社会资本参与的投资项目，主动与社会、企业合作，政府不唱“独角戏”，激发投资活力。坚持项目签约与落实责任同步、督促检查与解决问题同步、宣传推介与优化环境同步，提高签约项目履约率和资金到位率。全年实际利用市域外资金120亿元以上。

　　（二）坚持多元融合发展，加快推进经济转型

　　突出工业优化升级。坚持工业强市战略。改造提升传统产业，积极引进实施煤炭深加工项目，推动煤炭清洁、高效利用。支持鼓励建材企业到中亚国家释放优势产能，开发水泥新产品和节能环保型建材。加快铭帝铝材深加工项目建设，加强与知名陶瓷企业合作，力争在建筑陶瓷、日用陶瓷、高端工艺陶瓷发展上取得突破。大力发展装备制造、食品加工、医药保健等接续产业，抓好旺旺三期、四期项目建设。加快石油、紫砂土等资源开发，提高接续产业对经济的贡献份额。积极实施创新驱动战略，增强企业科技创新能力，推动信息化与工业化深度融合发展。

　　大力发展现代农业。坚决落实各项强农惠农富农政策，健全“三农”投资稳定增长机制。坚守耕地红线，全面开展永久基本农田划定，粮食播种面积85万亩，总产达到20万吨以上。稳定鲜干果经济林面积，提高管护水平，推进标准化生产。建设3个500头奶牛场和宜君肉牛养殖基地县。统筹推进照金（小丘）、步寿塬、黄堡、周陵、福地（棋盘）5个现代农业综合示范区建设。建成肉菜流通追溯体系，促进农村土地经营权有序流转，加快耀州区、宜君县职业农民塑造工程示范县建设。

　　扶持旅游产业发展。依托自然及文物文化资源，打造休闲养生旅游目的地城市。扎实推进与陕文投、陕煤化全域旅游合作，积极发展民宿体验、养生保健、休闲度假等旅游新业态。扩大照金效应，加快实施照金文化旅游名镇二期工程，建设照金红色教育基地。打响药王品牌，加快药王山景区建设，建成孙思邈纪念馆、游客服务中心及崇福阁等项目。加快玉华宫景区居民搬迁，启动玉华宫景前区建设，推进景镇（太安镇）融合。启动陈炉古镇核心区建设，打造耀瓷旅游精品线路。在新区建设文化旅游综合体。大力发展乡村旅游，打造马咀养生体验、申河香谷湿地、孟姜女故里游、王石凹煤矿工业遗址公园等旅游品牌，支持宜君县全域旅游发展。实施旅游项目30个，完成投资10亿元，旅游总收入增长15%。

　　（三）坚持统筹协调发展，提高城乡建设水平

　　强化规划引领约束。运用系统化思维，树立超前意识，立足优势互补、差异发展，在交通一体化、生态环保、休闲健康、产业升级转型方面，务实推进西铜同城化。紧扣我市在“一带一路”建设、关天经济区发展规划和关中城市群中的定位，启动《城市总体规划》修编工作，市区、景区一体，生活、休闲同步，推进新型城镇化。加强城市红线、绿线等刚性指标管理。重视城市设计和专项规划编制，强化建筑风格和色彩管控，彰显地域文化特色。规划为纲，规则为王，坚决查处不按规划设计施工等违规行为，增强规划执行力。

　　健全城市服务功能。创新投融资机制，吸引更多社会资金，加快城市基础设施建设。加强王益区、印台区片区改造提升，控建增绿，以业兴城，完善城市服务功能。突出形态开发和功能开发，加大新区开发建设力度，合理布局中小学校、社区医疗和商业网点，加快华原西道、华夏南道、西环线北段和北环线西段建设，九州东道建成通车，实施城市集中供热和排水防涝工程，建设长虹路地下综合管廊，实现速变巨变。推进耀州区统筹城乡发展试点，加快耀州古城改造和锦阳新城建设，促进新耀联动融合发展。加快宜君秀美山城建设，增强产业和人口聚集能力。继续抓好黄堡重点示范镇和照金、陈炉文化旅游名镇建设。深化品牌客运创建活动。

　　夯实精细管理责任。巩固提升国家卫生城市创建成果。继续推进城市亮化工程。理顺城市管理体制，加强综合执法队伍建设，提高执法和服务水平。坚持徒步街巷现场管理，建立标准化管理体系和巡查考评制度。建设智慧城市，用大数据推进全方位、实时有效服务和监管。加强城市社区建设，规范住宅小区物业管理，注重对背街小巷、城中村、城乡结合部的管理服务，把精细化、网格化管理落到人头、落到地面，确保没有空白、不留死角。

　　统筹精准扶贫开发。坚持美丽乡村建设与产业发展、农民增收与民生改善紧密结合，促进一、二、三产业融合发展，实施美丽乡村五大行动，加大对农村道路维护和垃圾、污水处理等基础设施建设的投入，以点带面、示范推进，加快2个部级、20个市级美丽乡村和10个新型农村社区建设。实施精准扶贫、精准脱贫，完成1.53万农村贫困人口脱贫和700户扶贫移民搬迁任务，一村一业、一村一特色，打造宜居、宜业、宜游的美丽乡村。建成县乡公路40公里、通村公路60公里。

　　促进农民工市民化。落实户籍制度改革政策，优先解决在城镇稳定就业和生活的常住人口落户问题，将随迁子女义务教育经费纳入财政保障范畴，将进城落户农民纳入城镇住房保障体系。实施农民工职业技能提升计划，让农民就业不离家、就地市民化。进一步完善农民工工资支付保障长效机制，扩大城镇社会保险对农民工的覆盖面，依法保障农民工合法权益，使农民工过上和城里人一样的生活。

　　（四）坚持绿色低碳发展，推进生态文明建设

　　综合施策治污降霾。严格落实《环境保护法》和铜川环保“铁十条”，坚持手段过硬、技术对路、措施到位，健全环境质量目标各级政府负责制。持续巩固治污降霾专项行动成果。完成华能照金电厂1号机组超低排放改造，加快启动华能铁路专用线建设。加大药王山周边环境整治。强化210国道、耀旬路等路域环境综合治理，严查运输车辆超速、超载、抛洒行为。严格落实建筑工地扬尘治理措施，全面拆除城市规划区天然气通达地段燃煤锅炉，城市建成区主干道机扫率达到90%以上，禁止露天焚烧秸杆和垃圾。新增天然气用户8000户。加强机动车尾气治理，加大客运车辆使用清洁燃料推广力度，淘汰黄标车及老旧机动车2326辆。对环境违法行为“零容忍”。全年空气质量优良天数达到200天以上。

　　持续用力改善生态。深入推进节能减排财政政策综合示范市创建工作，巩固提升全国绿化模范城市创建成果，完成省级环保模范城市创建年度任务。推进绿化铜川“五大工程”建设，全年植树造林12万亩，建成铜川植物园。开工建设印台后河沟水库和沮河生态工程，做好赵氏河国家湿地公园前期工作。启动新一轮全市地质灾害隐患点居民避险搬迁工程，做好废弃矿山环境恢复治理和复垦。治理水土流失150平方公里。开展饮水质量普查，加强饮用水源地保护，提升居民饮用水水质。设立农村饮水村级水质安全监督员，健全农村供水工程维修养护机制。加快实施污水处理厂提标改造工程，强化污染源在线监测，及时向社会公布监测结果，确保出市断面水质稳定达标。推进村容村貌整治，加强农村面源污染治理和环境保护。

　　节约集约利用资源。严格落实环境准入、资源环境总量控制、节能减排责任考核制度。全面推进工业、交通、建筑和公共机构节能。坚守水资源开发利用控制、用水效率控制和水功能区限制纳污“三条红线”，提高中水回用率，万元工业增加值用水量下降1%。建立节约集约土地利用评价考核机制，提高单位面积土地利用强度和投资强度，加大城乡低效闲置土地处置力度。完善资源有偿使用、生态补偿和损害赔偿制度，推动永续发展。

　　各位代表，生态环保一定要算大账、算长远账、算整体账、算综合账，关键在人努力，贵在行动、成在坚持。我们一定要天天抓、抓天天，天天看、看天天，空气质量一天不好都不行，一定要让铜川的天更蓝、地更绿、空气更清新！

　　（五）坚持改革开放创新，增强发展的新动能

　　落实行政改革举措。积极创建省级依法行政示范市。继续精简行政审批事项，简化审批事项办理流程。全面取消非行政许可审批，加快行业协会商会与行政机关脱钩，整治“红顶中介”。用政府权力的“减法”，换取市场活力的“乘法”。深入推进商事制度改革。规范政府债务管理，防范和妥善化解债务风险。抓好不动产统一登记工作。完成市、县、乡政府机构改革，分类推进事业单位改革，做好第九次村委会换届选举工作。完成公车使用制度改革。

　　深化经济体制改革。落实支持县（区）域经济发展6条措施，加大支持区县一切有利于发展的改革创新举措，让基层一线有人有权有钱能办事。深化国企国资改革，规范机关事业单位经营性资产管理，推进市属企业分类管理，支持非公经济参与国有企业重组改制，完成市纺织品公司改制，推进市燃气公司重组，组建市文化旅游投资控股公司，规范国有企业负责人薪酬和履职待遇。支持非公企业、中小微企业发展。加快居民用电户表改造步伐，实施居民用水、用电阶梯价格和公路客运价格改革。深化集体林权制度改革。深入推进“营改增”，加强资源税从价计征改革后的征管。建设“金惠工程”重点推广区，推进农信社向农商行转型，设立村镇银行，发展普惠金融。

　　推进社会领域改革。创新公共服务供给模式，加大政府向社会购买服务力度。深化文化体制改革，促进传统媒体与新兴媒体融合发展，构建现代文化市场体系和公共文化服务体系。深化教育领域综合改革，严格实行义务教育免试就近入学制度，逐步取消小学附设学前班，70%以上学校实施高效课堂。深化公立医院综合改革，逐步构建分级诊疗就医新秩序。

　　积极扩大对外开放。开放也是改革。抓住“一带一路”战略机遇，在产业转移、文化交流、对外贸易等方面主动出击，力争将玉华宫、宜君艾蒿洼娑罗古树纳入“丝路申遗”拓展项目。主动承接国内外产业转移，争取在向西开放的格局中有所作为。加强外贸人才培养和龙头企业培育，扩大苹果、陶瓷等地产品出口。全年外贸进出口总额2200万美元以上。积极主动邀请“一带一路”相关国家共同参与，精心策划、高质量办好第三届中国孙思邈中医药文化节。

　　（六）坚持顺应群众期盼，切实保障改善民生

　　鼓励创业促进就业。加强政府公共就业服务能力建设，积极创建省级创业型城市，使“草根”创新蔚然成风。认真落实促进就业创业各项政策措施，大力发展众创空间，形成大众创业、万众创新的生动局面。积极增加公益性岗位，向困难群众提供免费职业培训。做好高校毕业生、农村转移劳动力、城镇困难人员就业工作。全年发放小额担保贷款2亿元以上，城镇新增就业8000人以上。

　　抓好住房建设管理。将保障房建设与棚户区、城中村改造相结合，既按需建设，又注重通过市场筹集房源，提高保障效率。抓好锦绣新城配套设施建设，把“好房子、好环境、好服务”的要求落到实处，提高入住率。开工建设租赁型保障房3000套，棚户区改造7175户，基本建成各类保障房5000套，新增保障性住房租金补贴600户。

　　完善社会保障体系。启动机关事业单位养老保险制度改革。企业退休人员基本养老金标准提高10%，城乡居民基础养老金每人每月提高15元，城乡居民医保政府补助标准提高到人均不低于400元。城市低保标准每人每月提高35元，农村低保标准每人每年提高230元。建设13个城市社区服务体系项目。加快社会养老服务体系建设，持续投入办好农村幸福院，不断提高养老服务设施覆盖率。大力发展妇女儿童事业，加强未成年人保护。完善社会救助、社会福利服务体系，做好农村五保户、城市“三无”人员、高龄和失能老人、残疾人生活保障。支持志愿服务和慈善事业发展，让身处困境者都能及时得到社会关爱和温暖。

　　办人民满意的教育。加快实施第二期学前教育三年行动计划，将学前一年财政补助标准提高到生均每年1300元，新改扩建幼儿园11所。提升薄弱学校办学条件和水平，抓好王益区、宜君县“双高双普”创建和义务教育均衡发展。深入推进大学区管理，办好必要的村小学和教学点。加快普通高中特色化多样化、职业教育规模化实用化发展步伐，实施特殊教育三年提升计划，规范民办学校及校外补习机构办学行为。坚持立德树人，重视中小学生身心健康，稳步实施农村义务教育学生营养改善计划。资助400名家庭经济困难大学生，为6000名大学生提供生源地助学贷款。积极推进铜川职业技术学院二期工程建设。落实与北京中医药大学战略合作协议，加快北京中医药大学孙思邈学院建设。

　　提高医疗健康水平。优化医疗资源配置，加快市医院新区二期建设，部分投入使用，市中医院新区项目建成投运，逐步改善矿区和乡村医疗设施条件，加强乡村医生队伍建设。重视医德医风建设，构建和谐医患关系。推进居民医疗费用省内直接结算，深化药品供应保障体系改革，保障医疗机构临床用药需求和群众用药安全。抓好基本公共卫生服务项目精细化考核评价试点。推进第三轮全国艾滋病综合防治示范区建设。全面落实计划生育奖扶政策。

　　扎实推进文化建设。践行社会主义核心价值观，增强全民国防意识，弘扬铜川精神，深入开展省级文明城市创建工作。创建国家公共文化服务体系示范区。厚植创业创新文化，倡导全民阅读，推进文化惠民工程，政府购买文化演出300场。坚持设施建设和运行管理并重，加快补缺性、标志性文化工程和县、乡、村公共文化设施建设。实施重大文化艺术精品创作工程，广泛开展具有铜川特色的群众文化和全民健身活动，举办市第五届老年人运动会。开展大美铜川精彩故事系列主题宣传。加强耀州窑、玉华宫等遗址保护和利用，加快建设铜川博物馆。

　　提升社会治理水平。认真贯彻《安全生产法》，打造本质安全稳定型企业和行业。全面落实“党政同责、一岗双责、齐抓共管”的安全生产责任体系，加强煤矿、交通、非煤矿山、建筑施工、人员密集场所等重点领域的专项整治，坚决遏制重特大安全事故发生。全过程保障食品药品安全。强化防灾减灾预警体系建设，提升应急救援能力。促进宗教关系和谐。健全立体化社会治安防控体系，努力打造全省安全城市。深入开展法治宣传教育，建成区县和10个乡镇法律服务中心。严格落实信访工作责任，加大矛盾纠纷排查化解力度，依法依规解决群众合理诉求。

　　今年是“十二五”收官之年，我们要在完成“十二五”经济社会发展主要目标任务的同时，以改革的精神、创新的理念和科学的方法，做好“十三五”规划纲要编制工作，谋划好未来五年的发展蓝图。

　　四、加强法治政府建设

　　认真贯彻党的十八届四中全会精神，深入推进政府职能转变，加快建设职能科学、权责法定、执法严明、公开公正、廉洁高效、守法诚信的法治政府。

　　坚持严格依法行政。用法治思维和法治方式履行职责，全面落实行政执法责任制。自觉接受市人大法律监督和市政协民主监督，认真听取人大代表、民主党派、工商联、无党派人士和人民团体的意见。坚持法无授权不可为、法定职责必须为，简政放权、放管结合，公布权力清单、责任清单和负面清单，让权力在法治框架内运行，有权不可任性。所有行政行为都要于法有据，任何政府部门都不得法外设权。健全领导干部学法用法和政府法律顾问制度，建立行政机关内部重大决策合法性审查机制、重大决策终身责任追究制以及责任倒查机制。积极推进综合行政执法体制改革，着力解决权责交叉、多头执法、执法不严不正等问题，严格规范公正文明执法。

　　全面推进政务公开。坚持以公开为常态、不公开为例外。完善政务和各领域办事公开制度，推广电子政务和网上办事，做好政府信息发布和政策解读。扎实开展公民代表进政府和大学生假期到政府见习活动。高度重视网民留言办理和政务微博微信发布，创造条件让人民监督和批评政府。畅通公众意见反映和诉求听取渠道，重视舆情特别是网络舆情的收集和办理，发展和规范网络空间，广泛听取民声民意，及时认真回应社会关切。

　　不断改进工作作风。坚持纠正“四风”和树立新风并举，认真开展“三严三实”专题教育活动，深入开展“为民服务零距离”活动，巩固和拓展群众路线教育实践活动成果。严格落实中央八项规定、国务院“约法三章”，严控“三公”经费，加大对“四风”问题的查处力度。对为官不为、懒政怠政的，要公开曝光、坚决追究责任。新常态下要有新状态、应有新作为，不能用“老常态”对付“新常态”。下大气力解决本领恐慌、言行落后的问题，推进治理体系和治理能力现代化。强化担当和创新意识，发扬“钉钉子”精神，实打实现场推进各项重点工作。低调务实不张扬，埋头苦干抓落实，增强政府执行力和公信力。

　　切实加强廉政建设。以正风肃纪为抓手，以制度约束为重点，持续推进政府系统党风廉政建设和反腐败斗争。严守政治纪律和政治规矩，用制度形成对权力的有效制约，坚决打掉寻租空间，以权力瘦身为廉政强身。公职人员要始终敬畏人民，带头遵纪守法，自觉加强党性锻炼和公德家风修养，平时公道，关键时刻地道，不能出了事才明白自己不懂法。加强行政监察，加大领导干部经济责任审计力度。加大违纪违法案件查处力度，把严的责任和严的风气立起来，努力营造良好的从政环境和政治生态，在严于律己中担当开拓，在清风正气中干事创业，竭诚尽智为群众谋福祉。

　　各位代表！使命赋予责任，压力激发动力。让我们把思想和行动统一到习近平总书记来陕视察重要讲话精神上来，以党的十八大和十八届三中、四中全会精神为指引，在市委的坚强领导下，坚定信心、奋发有为，干在实处、走在前列，扎实做好今年的各项工作，把铜川发展得一年更比一年好！
